# Evaluating the impact of interprofessional training wards on patient satisfaction and clinical outcomes: a mixed-methods analysis

**DOI:** 10.3389/fmed.2024.1320027

**Published:** 2024-02-20

**Authors:** Sophie Schlosser-Hupf, Elisabeth Aichner, Marcus Meier, Sheila Albaladejo-Fuertes, Kirstin Ruttmann, Sophia Rusch, Bernhard Michels, Alexander Mehrl, Claudia Kunst, Stephan Schmid, Martina Müller

**Affiliations:** ^1^Department of Internal Medicine I, Gastroenterology, Hepatology, Endocrinology, Rheumatology, and Infectious Diseases, University Hospital Regensburg, Regensburg, Germany; ^2^Nursing Development Department of the Care Management Head Office, University Hospital Regensburg, Humboldt-Universität zu Berlin, Regensburg, Germany

**Keywords:** interprofessional education, interprofessional training ward, patient satisfaction, clinical outcome, healthcare education, collaborative care, quality of care, internal medicine

## Abstract

**Introduction:**

Interprofessional teamwork is pivotal in modern healthcare, prompting the establishment of interprofessional training wards since 1996. While these wards serve as hubs for optimizing healthcare professional collaboration and communication, research into patient outcomes remains notably sparse and geographically limited, predominantly examining patient satisfaction and sparingly exploring other metrics like mortality or self-discharge rates. This study seeks to bridge this gap, comparing patient outcomes in interprofessional training wards and conventional wards under the hypothesis that the former offers no disadvantage to patient outcomes.

**Materials and methods:**

We explored patient outcomes within an interprofessional student ward called A-STAR at a University Hospital from October 2019 to December 2022. Engaging with patients discharged between May 2021 and April 2022, we utilized digital and paper-based anonymous questionnaires, catering to patient preference, to gather pertinent data.

**Results:**

Analysis of outcomes for 1,482 A-STAR (interprofessional student ward) and 5,752 conventional ward patients revealed noteworthy findings. A-STAR patients tended to be younger (59 vs. 61 years, *p* < 0.01) and more frequently male (73.5% vs. 70.4%, *p* = 0.025). Vital clinical outcomes, such as discharges against medical advice, complication-driven readmissions, and ICU transfers, were statistically similar between groups, as were mortality rates (1.2% vs. 1.3%, *p* = 0.468). A-STAR demonstrated high patient satisfaction, underscored by positive reflections on team competence, ward atmosphere, and responsiveness to concerns, emphasizing the value placed on interprofessional collaboration. Patient narratives commended team kindness, lucid explanations, and proactive involvement.

**Discussion:**

This data collectively underscores the safety and reliability of patient care within training wards, affirming that patients can trust the care provided in these settings. Patients on the interprofessional ward demonstrated high satisfaction levels: 96.7% appreciated the atmosphere and conduct of ward rounds. In comparison, 98.3% were satisfied with the discussion and information about their treatment during their hospital stay.

## 1 Introduction

Effective interprofessional teamwork is fundamental in modern healthcare management, necessitating interprofessional education (IPE) integration into health profession curricula, as widely acknowledged in the literature (1–9). IPE encompasses the collaborative learning and interaction among students and professionals from diverse medical disciplines. Various tools and teaching strategies, such as simulation-based education and rotations in rural and community settings, have been identified for IPE implementation (10). Originating in Scandinavia in 1996 and subsequently globalized (11–17), interprofessional training wards within hospitals serve as multifaceted training hubs. Aspiring professionals from various healthcare disciplines, including medicine, nursing, pharmacy, and physiotherapy, autonomously deliver patient care under supervisory guidance, focusing on delivering medical excellence in patient care and fostering optimal medical collaboration (18–20).

There are already several studies that demonstrate the high value of interprofessional training wards for interprofessional education and gain of interprofessional competence (12, 17, 21–35): Brätz et al. found that final-year medical students who received training on an interprofessional training ward (ITW) showed significantly higher entrustment scores for 10 of 12 entrustable professional activities compared to a control group (25). The most significant variances and impacts were observed in relation to “Engagement with a consultant” and “Conducting a presentation about an oncology patient in a tumor board meeting.” These two aspects have demonstrated a strong correlation with the competency aspect of “Oral communication with colleagues and supervisors”. Mink et al. documented statistically significant positive impacts, both short-term and long-term, of interprofessional education within clinical settings on students’ perceptions of interprofessional socialization and teamwork (27). Specifically, participants in the interprofessional training ward IPAPAED exhibited enhanced scores in interprofessional socialization and valuation, alongside improved communication skills and adaptability within interprofessional teams. Notably, these improvements persisted over a 6 to 34-month period, indicating the enduring positive effects of interprofessional learning and collaboration within the IPAPAED framework (24). Freeth et al. highlighted the high value placed by students on “real life” clinical experiences (35), while Morphet et al. noted a positive learning environment and high satisfaction rates among students, correlating with enhanced teamwork and collaborative skills (31). Gender-related differences in perceptions of the value of Interprofessional Training Wards (IPTW) were observed, with female students exhibiting slightly more positivity compared to male students in the study by Lindh Falk et al. (30).

These studies underscore the critical role of interprofessional education in enhancing collaborative skills, communication competencies, and professional entrustment in healthcare. It is hypothesized that interprofessional training wards not only enhance individual student learning but also contribute to the overarching goal of delivering superior patient care through effective teamwork and collaboration across various healthcare disciplines.

Ensuring excellent education for future medical professionals without compromising patient care is paramount. Numerous studies have explored interprofessional interventions and their impacts on patient outcomes (36–53), revealing that interprofessional collaboration leads to significant improvements in patient outcomes. Despite criticisms regarding methodological rigor, as noted in Lutfiyya et al.’s literature review (49), the named studies suggest that interprofessional education can have a positive impact on patient outcomes, including patient safety, quality of life, and functional status as described in the latest literature review of Purnasiwi et al. (51). However, Dow et al. cautioned against the complexities inherent in interprofessional networks that may pose challenges to effective collaboration (54).

Research specifically investigating patient outcomes within interprofessional training units remains limited (13, 16, 35, 55–65), primarily emanating from Europe (13, 35, 38, 39, 55–59, 61, 62, 66) and Australia (16, 60). Predominantly located in surgical (13, 58) and orthopedic departments (35, 55–57, 61, 66), interprofessional training wards in other specialties like general medicine (16), emergency medicine (60), pediatrics (62), and rheumatology (35, 61) are underexplored. Existing studies often prioritize qualitative evaluations of patient satisfaction (13, 16, 19, 35, 56, 57, 59–62, 66, 67), with quantitative research typically focusing on complications (19, 38–41, 55, 57, 58) and sparingly on mortality (55, 58). Notably, data regarding discharges against medical advice remain absent.

The overarching aim of this research is to ascertain whether and how IPE contributes to more effective healthcare teams and delivers better patient care. Our study examined potential disparities in patient outcomes between interprofessional training wards and conventional wards, both quantitatively and qualitatively, reflecting the complexity of evaluating educational interventions in dynamic real-world healthcare settings. Kirkpatrick’s Evaluation Model, commonly employed in evaluating interprofessional training programs (68, 69) assesses the impact of interprofessional training on students’ attitudes (reaction), knowledge and skills (learning), professional behavior (behavior), and patient outcomes (results). Our study specifically focused on the last aspect of Kirkpatrick’s Evaluation Model “results,” operating under the hypothesis that interprofessional training wards do not compromise patient outcomes. We meticulously examined patient satisfaction, perceived team competence, mortality, complications leading to readmission or ICU transfer, and discharges against medical advice on our interprofessional training ward A-STAR.

## 2 Materials and methods

### 2.1 Patients

Patients admitted to the A-STAR interprofessional ward and conventional wards within the Department of Internal Medicine I at University Hospital Regensburg (October 1, 2019 – December 31, 2022) were considered for quantitative outcome parameter analysis. To account for annual closures from December 23rd to January 1st, admissions and discharges from December 20th to January 6th were systematically excluded to mitigate selection bias. Patients discharged from May 1, 2021, to April 30, 2022, were provided an anonymous questionnaire, available in both online and paper formats (Questor Pro 5, Blubbsoft GmbH, Berlin), with details available in Supplementary material S1.

### 2.2 Trial design

This monocentric, open-label, controlled study employed no formal randomization but utilized case managers who were uninvolved in the study to allocate patients to the A-STAR or conventional wards randomly. Ethical approval was granted by the University of Regensburg’s Ethics Committee (20-1805_1-101), and the study adhered to the latest Declaration of Helsinki, the International Conference on Harmonisation’s Good Clinical Practice guidelines, and pertinent German regulations.

### 2.3 Understanding the application process: interprofessional training ward enrollment and clinical work on the interprofessional student ward

The WHO Framework for Action on Interprofessional Education & Collaborative Practice and other national and international interprofessional education (IPE) competencies and frameworks played a pivotal role in shaping both the conception of the A-STAR and the selection of an appropriate evaluation approach (1, 70–87).

Conventional ward care was administered by medical professionals and nurses, with added support from final-year medical students and nursing trainees. The interprofessional A-STAR unit was managed per shift by a team of up to six senior medical students and two 2nd and 3rd-year nursing trainees, all under the supervision of experienced healthcare staff. A-STAR team selection hinged on a review of applicants’ motivation letters and comprehensive CVs by the department head and nursing team lead. Medical students devoted 8–16 weeks of their final year to the unit, while nursing trainees participated for approximately 4 weeks. Medical students had previously completed all theoretical and medical courses of their medical studies and the second of three final exams. The nursing trainees had completed at least 1 year of their total 3-year training.

Integrating seamlessly into conventional wards, the A-STAR unit fostered collaborative care by uniting medical students and nursing trainees in a shared base, whereas doctors and nurses in conventional wards operated from distinct bases. With an 8- to 12-bed capacity, the A-STAR unit contrasted with the conventional wards’ 45- to 49-bed capacity. Both ward types catered to a diverse patient population, addressing gastroenterological, hepatological, infectious, endocrine, and rheumatological conditions.

The A-STAR unit adhered to a structured daily routine, encompassing planning sessions, patient visits, educational interactions, and feedback dialogues. Days were initiated with a joint interprofessional plan after the night shift’s nursing handover, followed by routine tasks executed by nursing trainees. Medical students and nursing trainees jointly conducted ward rounds, a practice mirrored by doctors and nurses in conventional wards. Pharmacology students, pharmacists, physiotherapists, and nutritionists contributed to A-STAR rounds every week, scrutinizing medication interactions and dosages, while conventional wards received weekly pharmacy counsel for specific cases. The conventional wards worked in the traditional way and for the most part did not have joint medical and nursing rounds. All wards benefited from daily educational visits led by a medical director or a senior medical representative. The supervising healthcare staff of the A-STAR only intervened in the event of patient-endangering behavior and treated the trainees like young professionals.

The A-STAR unit integrated medical students and nursing trainees in daily, multifaceted, interprofessional educational sessions enhanced by the expertise of varied healthcare professionals. Its inclusive training curriculum featured specialty training—spanning resuscitation, hands-on skills via models, and in-depth sonography courses—ensuring a holistic educational curriculum. This interprofessional and interdisciplinary collective, including pharmacists, physiotherapists, nutritionists, chaplains, technicians, and psychologists, gathered daily for interdisciplinary discussions and X-ray presentations, with active participation from medical students and nursing trainees. Days ended with feedback and reflective discussions, solidifying the unit’s educational and collaborative approach.

### 2.4 Evaluating patient outcomes and resource utilization in healthcare settings

We examined critical patient outcomes and resource allocation through two primary lenses: mortality and Case-Mix-Index (CMI). Secondary outcomes of focus included instances of discharge against medical advice, complications, and transfers to the Intensive Care Unit (ICU). The CMI, prevalent in German healthcare, numerically represents the average resource intensity, acknowledging diverse factors like diagnoses and procedures during a patient’s stay, thus symbolizing the respective care level and resources. A higher CMI suggests that patients necessitate enhanced medical attention and resource deployment. Data were procured from the hospital patient register.

Patients discharged from May 1, 2021, to April 30, 2022, were invited to contribute via an anonymous questionnaire, available online and in a paper-based format, per individual preference. The questionnaire is accessible in Supplementary material S1. In the absence of a standardized questionnaire during the study planning phase, we utilized the patient questionnaire from the clinic’s quality management, supplemented with inquiries on interprofessional core competences, aligned with international frameworks (1, 70, 71, 80, 85). The questionnaire, comprising 42 questions, addresses various aspects. The initial section comprises 21 questions assessing general stay information on a 5-point Likert scale. Subsequent sections evaluate organization, examinations, and nursing measures using a 5-point Likert scale. Following this, three sections gauge knowledge and competence, communication, professional appearance, and empathy of medical and nursing students and professional staff on a 5-point Likert scale. The questionnaire concludes with queries on health status, two open-ended general feedback questions, and categorization based on age group and length of stay. Prior to implementation, the questionnaire underwent face validation in a small patient cohort.

### 2.5 Statistical analysis – statistical comparison of qualitative variables

Qualitative variables underwent comparative analysis utilizing Pearson’s chi-square test of independence. All tests were two-sided, with a *p*-value under 0.05 denoting statistical significance. Analyses were conducted using IBM SPSS Statistics for Windows, version 28.0 (released in 2021) by IBM Corp., Armonk, NY.

## 3 Results

Of the respondents, 281 completed the questionnaire, achieving an 84.6% response rate. This included 125 patients from the A-STAR and 156 from conventional wards. Table 1 illustrates the participants’ sociodemographic data, revealing no significant group discrepancies.

**Table 1:** Patient characteristics of qualitative outcome analysis (2021–2022).

Characteristic	A-STAR (*n* = 125)	Conventional wards (*n* = 156)	
Age			χ^2^(4) = 2.73; *p* = 0.604
18 to 24 years	8.0% (10)	4.5% (7)	
25 to 34 years	6.4% (8)	7.1% (11)	
35 to 50 years	14.4% (18)	11.5% (18)	
51 to 70 years	50.4% (63)	51.3% (80)	
Above 70 years	13.6% (17)	17.9% (28)	
NA	7.2% (9)	7.7% (12)	
Length of stay			χ^2^(3) = 4.55; *p* = 0.208
1 to 3 days	27.2% (34)	22.4% (35)	
4 to 7 days	32.0% (40)	25.0% (39)	
8 to 14 days	20.8% (26)	26.9% (42)	
Above 14 days	10.4% (13)	16.0% (25)	
NA	9.6% (12)	9.6% (15)	
Subjective health level at admission			χ^2^(3) = 5.85; *p* = 0.119
Very good	34,4% (43)	28.2% (44)	
Rather good	24.0% (30)	28.2% (44)	
Rather poor	25.6% (32)	21.2% (33)	
Very poor	10.4% (13)	19.9% (31)	
NA	5.6% (7)	2.6% (4)	
Subjective health level on the day of the interview			χ^2^(3) = 5.85; *p* = 0.500
Very good	45.6% (57)	38.5% (60)	
Rather good	40.0% (50)	49.4% (77)	
Rather poor	6.4% (8)	5.8% (9)	
Very poor	0.8% (1)	1.3% (2)	
NA	7.2% (9)	5.1% (8)	

### 3.1 Optimized patient interaction and communication in the interprofessional training ward A-STAR

**Figure 1:**
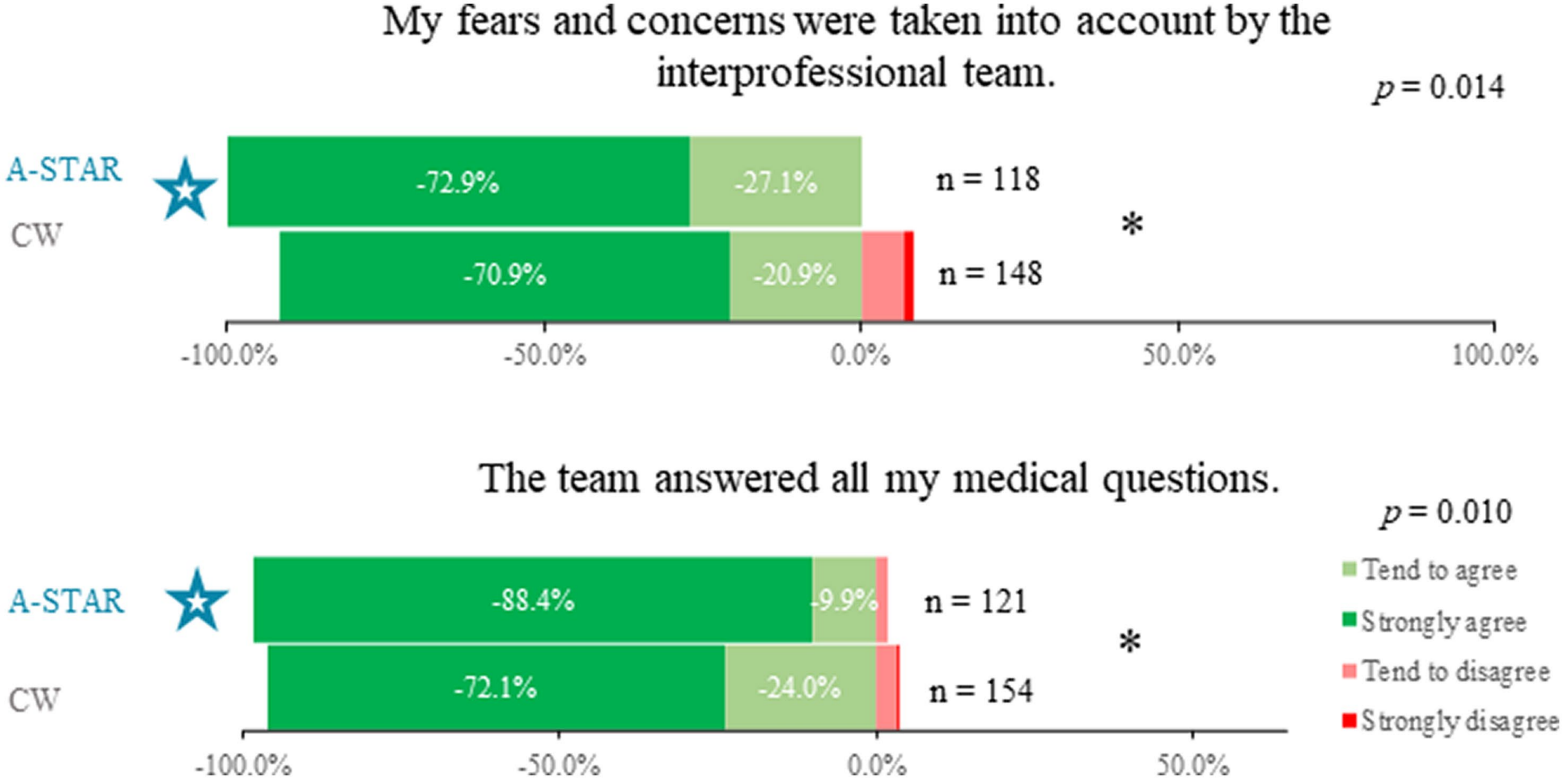
Enhanced patient interaction and query resolution in A-STAR vs. conventional wards. Patients in the A-STAR interprofessional training ward (*n* = 118) experienced more consideration of their fears and concerns compared to those in conventional wards (*n* = 148), (χ^2^(3) = 10.66; *p* = 0.014; φ = 0.203). Moreover, a significantly larger proportion of A-STAR patients (*n* = 121) reported having all their medical questions addressed by the team, in contrast to patients in the conventional wards (*n* = 154), (χ^2^(3) = 11.32, *p* = 0.010, φ = 0.200).

In addressing fears and concerns, patients in the A-STAR group felt more acknowledged by the interprofessional team compared to those in conventional wards (100% vs. 91.8%, χ^2^(3) = 10.66, *p* = 0.014, φ = 0.203, Figure 1). Furthermore, a higher percentage of A-STAR patients noted that the team addressed all their medical questions compared to the conventional wards (98.3% vs. 96.1%, χ^2^(3) = 11.32, *p* = 0.010, φ = 0.200, Figure 1).

### 3.2 High patient satisfaction in the interprofessional training ward A-STAR

**Figure 2:**
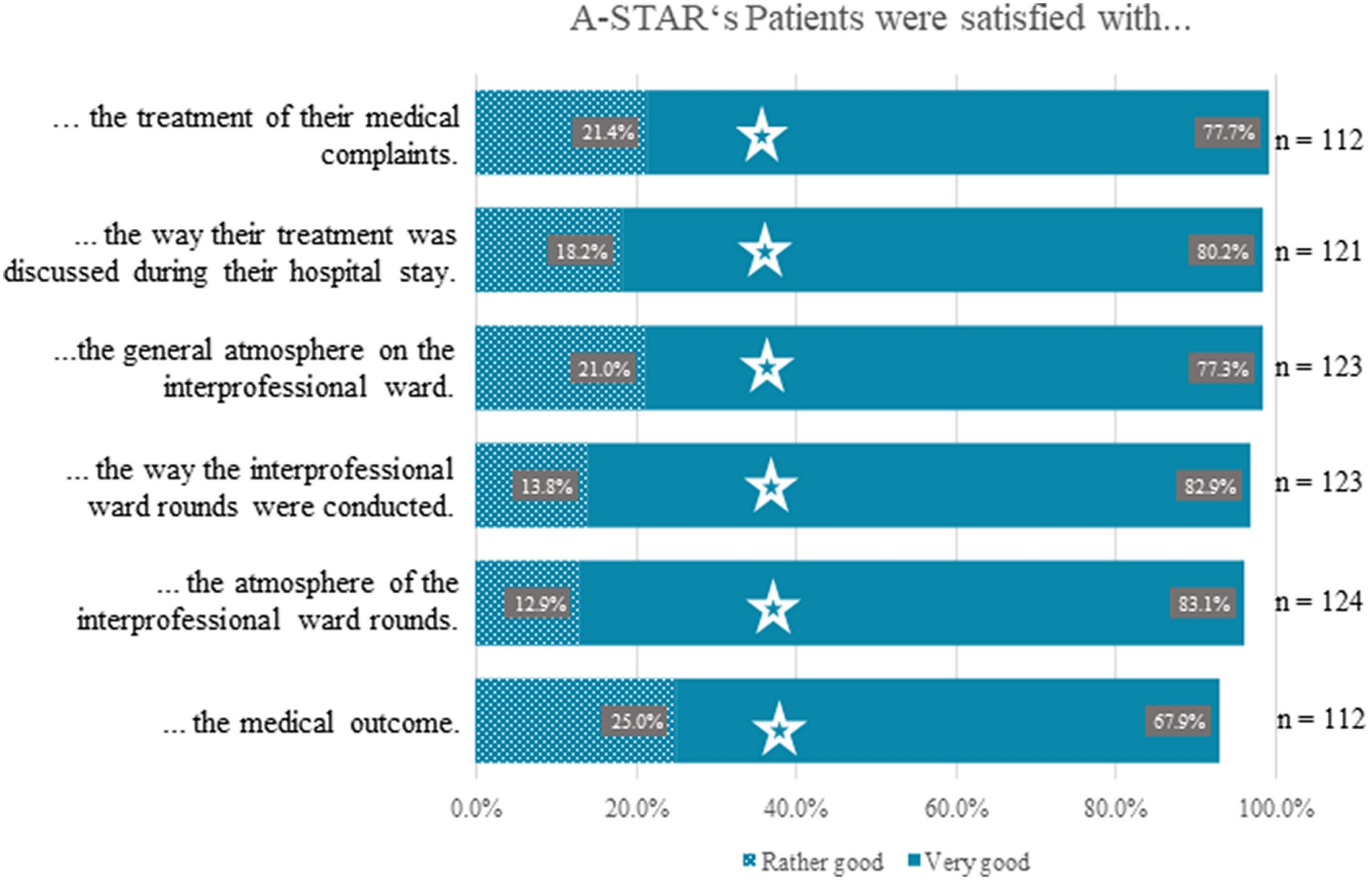
High levels of patient satisfaction across various care aspects in A-STAR interprofessional training ward. Patients in the A-STAR training ward expressed substantial satisfaction across multiple facets of their care and ward rounds. Satisfaction levels in these areas showed no significant variance compared to conventional wards, with equally excellent results across all ward types. Specifically, 99.1% (*n* = 112) were satisfied with the treatment of their medical complaints (χ^2^(3) = 3.43, *p* = 0.330), 98.4% (*n* = 121) appreciated discussions about their treatment during the stay (χ^2^(3) = 5.87, *p* = 0.118), and 98.3% (*n* = 123) endorsed the general atmosphere on the ward (χ^2^(3) = 3.41, *p* = 0.333). Additionally, satisfaction levels remained high concerning how interprofessional ward rounds were conducted (96.7%, *n* = 123; χ^2^(3) = 5.39, *p* = 0.146) and their atmosphere (96.0%, *n* = 124; χ^2^(3) = 1,814, *p* = 0.612). Furthermore, 92.9% (*n* = 112) were content with the medical outcome and acknowledged collaborating with the interprofessional medical team to decide on further care (χ^2^(3) = 1.97, *p* = 0.580). Notably, these results mirrored the perceptions from conventional wards, as evidenced by the Chi-Square test.

Patients in the A-STAR training ward expressed high satisfaction across various aspects of their care and ward rounds (Figure 2). Satisfaction rates in the following aspects were not significantly different from those in conventional wards. The results are comparably excellent regardless of the type of ward: Specifically, 99.1% (*n* = 112) were content with the treatment of their medical complaints (χ^2^(3) = 3.43, *p* = 0.330), 98.4% (*n* = 121) valued discussions about their treatment during the stay (χ^2^(3) = 5.87, *p* = 0.118), and 98.3% (*n* = 123) endorsed the overall atmosphere on the ward (χ^2^(3) = 3.41, *p* = 0.333). Additionally, satisfaction levels remained high regarding the conduct of interprofessional ward rounds (96.7%, *n* = 123; χ^2^(3) = 5.39, *p* = 0.146) and their atmosphere (96.0%, *n* = 124; χ^2^(3) = 1,814, *p* = 0.612). Furthermore, 92.9% (*n* = 112) were satisfied with the medical outcome and acknowledged collaborating with the interprofessional medical team to decide on further care (χ^2^(3) = 1.97, *p* = 0.580). Due to the comparable results, the figure for clarity only shows the outcomes from the interprofessional training ward (Figure 2).

### 3.3 Perceived professional competence: equivalence between A-STAR and conventional ward medical teams as viewed by patients

**Figure 3:**
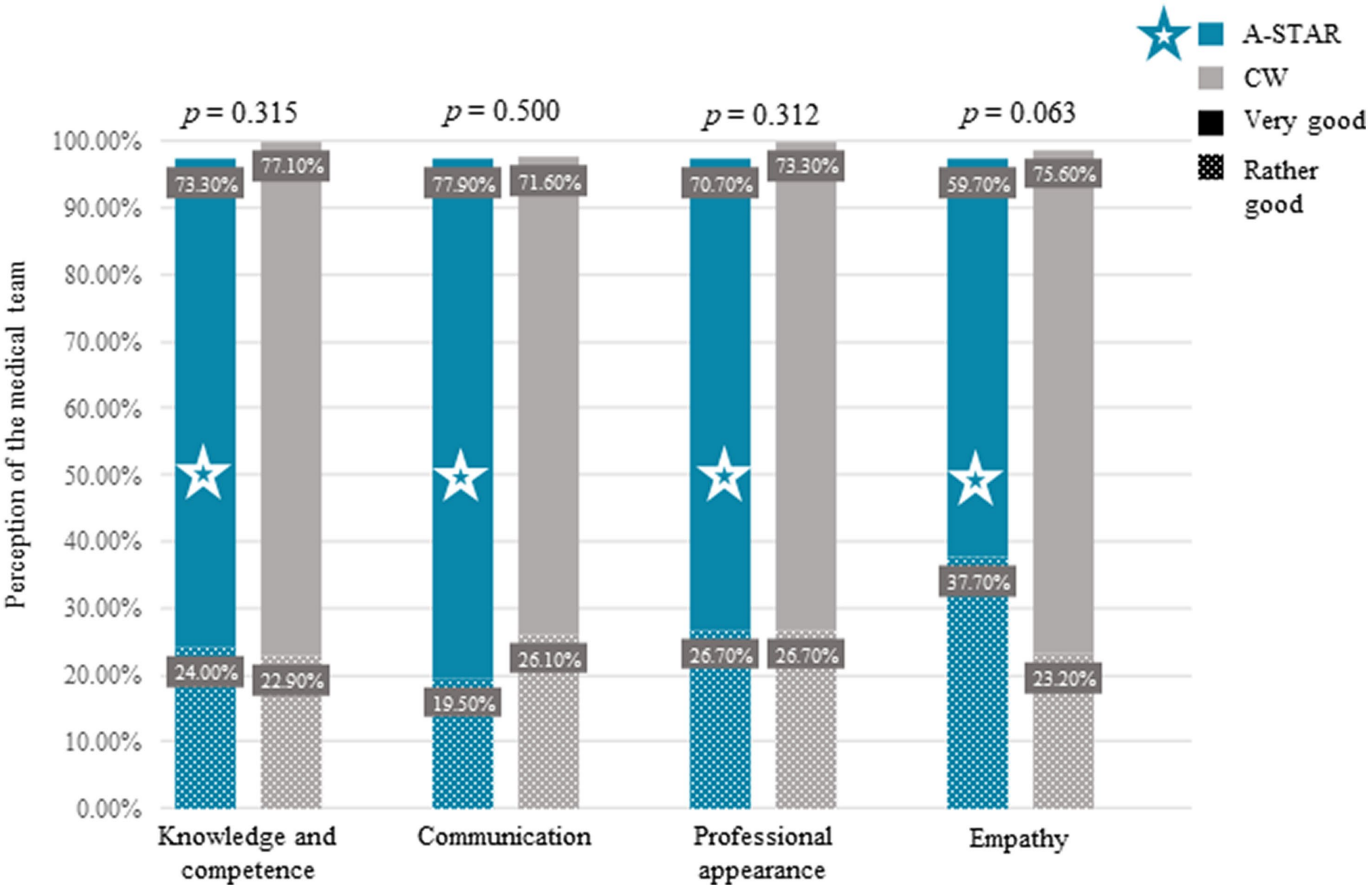
Comparative patient perceptions of medical team competency: A-STAR vs. conventional wards. Patients generally perceived medical teams in the A-STAR interprofessional training ward—led by final-year medical students and nursing trainees—as equivalently competent to the professional teams in conventional wards (CW), which professional doctors and nurses led. Notably, no significant differences were found in patient evaluations between the two ward types in terms of: Knowledge (A-STAR: *n* = 75, CW: *n* = 83; χ^2^(3) = 2.31. *p* = 0.315), communication (A-STAR: *n* = 77, CW: *n* = 88; χ^2^(3) = 2.37, *p* = 0.500), professional appearance (A-STAR: *n* = 75, CW: *n* = 86; χ^2^(3) = 2.33, *p* = 0.312), and empathy (A-STAR: *n* = 77; CW: *n* = 82; χ^2^(3) = 7.30, *p* = 0.063), as determined by the Chi-Square test.

Patient perceptions of competence were comparable between the A-STAR’s team of medical students and nursing trainees and the conventional wards’ professional medical teams in areas of knowledge (A-STAR: *n* = 75, CW: *n* = 83; χ^2^(3) = 2.31, *p* = 0.315), communication (A-STAR: *n* = 77, CW: *n* = 88; χ^2^(3) = 2.37, *p* = 0.500), professional demeanor (A-STAR: *n* = 75, CW: *n* = 86; χ^2^(3) = 2.33, *p* = 0.312), and empathy (A-STAR: *n* = 77, CW: *n* = 82; χ^2^(3) = 7.30, *p* = 0.063), as demonstrated in Figure 3. Both teams were highly rated across these domains.

### 3.4 Comparing patient outcomes: mortality and complications in A-STAR vs. conventional wards

We analyzed outcome data from 1,482 A-STAR group patients and 5,752 conventional ward patients, with sociodemographic details presented in Table 2. The A-STAR group had a notably higher percentage of male patients (73.5%) than the conventional wards (70.4%, χ ^2^(1) = 5.12, *p* = 0.024). Additionally, A-STAR patients were significantly younger, averaging 59 years, compared to the 61-year average in conventional wards (Mann–Whitney-U-Test, *p* < 0.01).

**Table 2:** Patient characteristics of the patients included in the outcome analysis (2019–2022).

Characteristic	A-STAR (*n* = 1,482)	Conventional wards (*n* = 5,752)	
Age			*p* < 0.01
Median (range) – yr	59 (18–101)	61 (16–98)	
Sex			χ^2^(1) = 5.12; *p* = 0.024
Male – no. (%)	1,089 (73.5)	4,052 (70.4)	
Female – no. (%)	393 (26.5)	1700 (29.5)	
CMI (2019–2022)	2.4	2.1	

**Figure 4:**
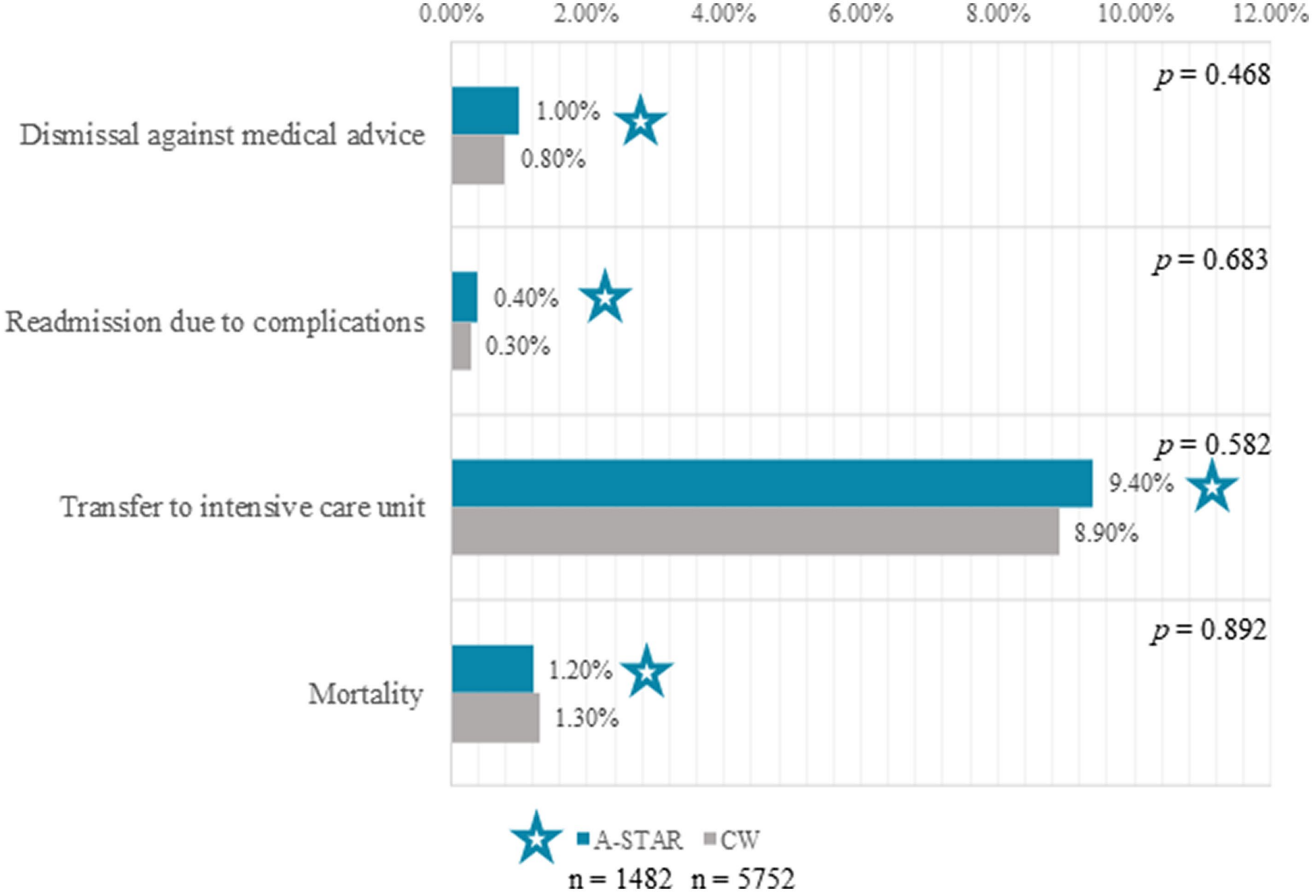
Clinical outcomes exhibited in A-STAR interprofessional training (*n* = 1,482, star) ward patients (*n* = 1,482, star) as compared to conventional wards (CW, *n* = 5,752). No significant difference was observed between the A-STAR group and conventional wards regarding discharge against medical advice (1.0% [*n* = 14] vs. 0.8% [*n* = 47]; χ^2^(1) = 0.53, *p* = 0.468). Comparable readmission rates due to complications were experienced in both groups (0.4% [*n* = 5] vs. 0.3% [*n* = 17]; χ^2^(1) = 0.17, *p* = 0.683). Patient transfers from both A-STAR and conventional wards to the intensive care unit were not significantly different (9.4% [*n* = 125] vs. 8.9% [*n* = 496]; χ^2^(1) = 0.30, *p* = 0.582). Mortality rates exhibited no significant divergence between the A-STAR group and conventional wards (1.2% [*n* = 16] vs. 1.3% [*n* = 71]; χ^2^(1) = 0.05, *p* = 0.892).

In examining patient outcomes, the A-STAR group and conventional wards demonstrated no significant differences in several key areas despite varying patient demographics and illness severities. Detailed findings, visualized in Figure 4, are outlined below:

- Discharge Against Medical Advice: Both groups presented similar occurrences of discharge against medical advice (A-STAR: 1% [*n* = 14] vs. conventional wards: 0.8% [*n* = 47], χ^2^(1) = 0.53, *p* = 0.468).
- Readmission Rates: Comparable rates of readmission due to complications were noted between A-STAR and conventional wards (0.4% [*n* = 5] vs. 0.3% [*n* = 17] respectively, χ^2^(1) = 0.17, *p* = 0.683).
- Transfer to Intensive Care: Transfer rates to intensive care units showed no significant difference between the two groups (A-STAR: 9.4% [*n* = 125] vs. Conventional Wards: 8.9% [*n* = 496], χ^2^(1) = 0.30, *p* = 0.582), even with the A-STAR group hosting patients with a higher average severity of illness (Case-Mix-Index: 2.4 vs. 2.1).
- Mortality Rates: Mortality rates were likewise consistent between A-STAR and conventional wards (1.2% [*n* = 16] vs. 1.3% [*n* = 71], χ^2^(1) = 0.05, *p* = 0.892). In both settings, 3/4 of the mortality cases were due to palliative conditions, including but not limited to cancer and acute-on-chronic liver failure.

### 3.5 Maximized patient engagements and positive views on care and cooperation within the A-STAR ward

The A-STAR as well as the conventional wards show notable results highlighting the positive impact of teamwork. The A-STAR demonstrates comparable outcomes to those of the conventional wards in the following aspects:

100% (*n* = 121) affirmed that the team assisted them in comprehending all pertinent information (χ^2^(3) = 5.10, *p* = 0.165),
99.2% (*n* = 123) felt well cared for (χ^2^(3) = 1.78, *p* = 0.411),
98.3% (*n* = 119) would recommend the ward to friends or family (χ^2^(3) = 2.19, *p* = 0.534),
97.4% (*n* = 114) believed the team understood what was important to them (χ^2^(3) = 3.53, *p* = 0.317), and
96.6% (*n* = 117) acknowledged the team’s collaboration as a positive influence on their well-being (χ^2^(3) = 6.55, *p* = 0.088).

**Figure 5:**
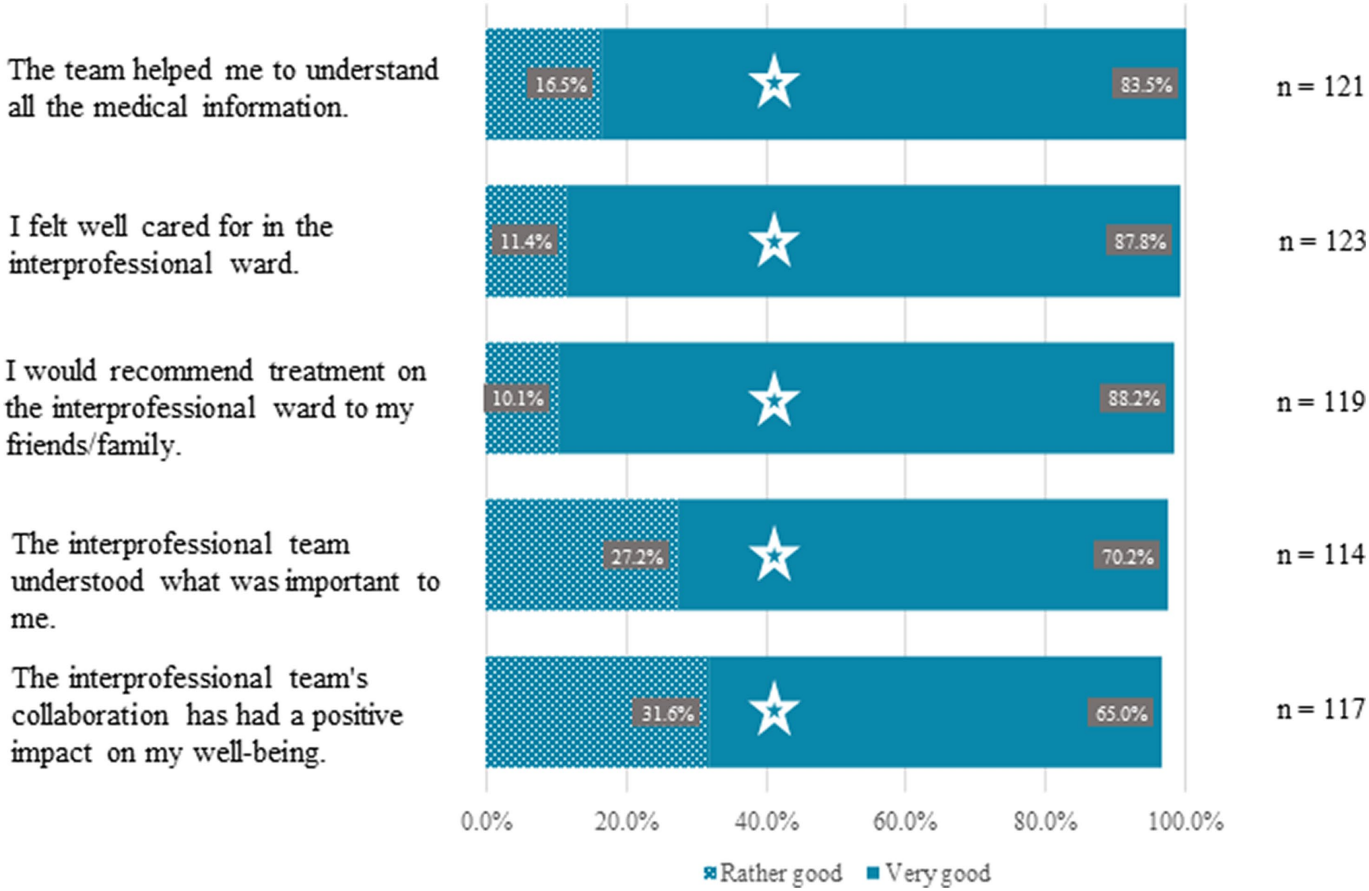
Optimized patient experiences and favorable perceptions of care and collaboration in the A-STAR Ward. A substantial majority of A-STAR patients positively reviewed their care, comparable to those of conventional wards: 96.6% (*n* = 117) affirmed the team’s collaboration beneficially impacted their well-being (χ^2^(3) = 6.55, *p* = 0.088); 97.4% (*n* = 114) felt the interprofessional team grasped their priorities (χ^2^(3) = 3.53, *p* = 0.317); 98.3% (*n* = 119) would recommend the interprofessional ward to friends/family (χ^2^(3) = 2.19, *p* = 0.534); 99.2% (*n* = 123) felt well cared for (χ^2^(3) = 1.78, *p* = 0.411); and 100% (*n* = 121) agreed the team facilitated understanding of medical information (χ^2^(3) = 5.10, *p* = 0.165).

These findings were statistically akin to perceptions from conventional wards, as indicated by the Chi-Square test results. The figure exclusively presents the results from the A-STAR (Figure 5).

### 3.6 Elevating patient experience through compassionate and inclusive care in A-STAR

Patients consistently lauded “the universal kindness of the staff in their open-ended feedback.” One patient spotlighted the clarity and comprehensiveness with which medical students explained medical aspects, appreciating the nursing staff’s availability and promptness in addressing problems or inquiries. Highlights from patient insights included:

- Intensive care and candid discussions regarding risks, potential outcomes, and therapeutic alternatives by medical students,
- Meticulous preparation for potentially severe scenarios, and
- Provisions for private, post-round discussions to address lingering questions.

Another patient valued the “prompt information and active involvement in the therapeutic journey,” acknowledging the feeling of being treated as an equal and active participant due to the unhurried, attentive interactions with staff. “It seemed like all team members, spanning medical to nursing, were acquainted with my symptoms and worries. Every professional group collaborated flawlessly, each playing their specific role,” shared another patient.

## 4 Discussion

### 4.1 Enhanced health outcomes through interprofessional collaboration: a scientific exploration

In 2009, a Cochrane review substantiated that interventions fostering practice-based interprofessional collaboration can ameliorate health-related processes and outcomes (67). However, deducing generalizable conclusions regarding interprofessional collaboration’s fundamental elements and efficacy proved complex due to constraints such as the scarcity of studies, variable sample population sizes, challenges in collaboration conceptualization and measurement, and variability in interventions and context (67). After this, the body of research has seen modest augmentation, with a predominant focus on exploring patient satisfaction (13, 16, 19, 35, 60–62, 66, 67). Typically, these studies have been constrained by a restricted case pool, and only a minimal subset has undertaken comparative analyses between training and conventional wards.

Our research stands out as the most comprehensive controlled study to date, meticulously examining patient satisfaction through a detailed 42-question survey. Our findings highlight that patient care on the A-STAR ward is not only on par with, but in certain aspects, exceeds the quality observed on conventional wards, both from subjective and objective standpoints.

Patients in our study uniformly expressed significant satisfaction across diverse facets of their hospital stay. In particular, they strongly appreciated the ward’s round atmosphere, its conduct, the clarity of information regarding their treatment and disease progression, and in-depth treatment discussions throughout their stay. Notably, a higher percentage of A-STAR group patients felt their medical questions and anxieties were thoroughly and considerately addressed by the healthcare team compared to those in conventional wards.

High patient satisfaction rates, especially in communication, resonate with findings from earlier studies in training ward environments. Freeth et al. pioneered patient satisfaction research in their UK rheumatology-orthopedic training ward, discovering a pronounced appreciation for enhanced attention among a cohort of 34 patients (35). A follow-up study by the same team compared patient experiences with conventional wards reinforced these insights, showing elevated satisfaction in areas like “patient question response,” “patient information provision,” and “meeting patient needs” (61). Lindblom et al. explored the satisfaction levels of almost 300 patients at their Swedish orthopedic training ward, surveying after medical care by students at varied educational levels (66). Patient feedback from their satisfaction questionnaire revealed prominent satisfaction, rated from good to excellent, particularly regarding disease and treatment information. The team’s interaction with and accessibility to patients was also highly valued. A study by Brewer et al. indicated similarly high satisfaction scores within their general medical training ward at Royal Perth Hospital in Australia, registering top-average scores in all categories of the hospital’s standard patient satisfaction survey (16). However, this study did not provide a comparison with conventional wards. In another investigation, Straub et al. evaluated satisfaction among 56 pediatric patients and 109 parents in a German pediatric training ward, finding exemplary ratings in information dissemination, interprofessional cooperation, and the influence of trainee nurses and physicians on overall care (62). Hallin et al., researching 84 patients in a Danish orthopedic training ward, found that patients felt significantly better informed and more satisfied regarding home care information (56). These patients also believed that the interprofessional training ward staff incorporated their home circumstances more comprehensively into discharge planning and reported a notably higher level of involvement in treatment decisions.

A limitation of our study involves using a non-standardized patient questionnaire. Conversely, Marcussen et al. applied the standardized Client Satisfaction Questionnaire (CSQ-8) in their study with 129 patients at a psychiatric training ward in Denmark, discovering significantly elevated satisfaction rates compared to a control group of 123 patients (59).

The consistently high patient satisfaction in interprofessional training wards can be ascribed to an optimal patient-healthcare-worker ratio, enabling more regular discussions and continual interprofessional briefings among healthcare workers, ensuring unified information distribution to the patient.

Our study is the first to compare trainee and professional healthcare team performances, revealing that A-STAR patients perceived medical students and nursing trainees as equally proficient as professional teams in conventional wards regarding knowledge, communication, professional demeanor, and empathy. Given patients’ constrained capacity to evaluate medical treatments due to their non-specialist knowledge, easily assessable aspects like communication become vital in enhancing their comfort and assurance in the care provided.

Furthermore, our study validates patient and health worker relationship perceptions in interprofessional training wards using the objective metric of discharges against medical advice. No significant differences were observed between the A-STAR group and conventional wards in this regard, indicating that trainees typically established effective doctor-patient relationships in most cases.

Despite A-STAR patients exhibiting a higher severity of illness, they experienced readmission rates and patient transfers to the intensive care unit comparable to those in conventional wards. While numerous studies illustrate that interprofessional interventions can reduce complication rates (19, 38–41, 55, 57, 58), data specifically on interprofessional training wards remain sparse. For example, Hallin et al. found no difference in 90-day readmission rates between their orthopedic training ward (1,109 patients) and conventional wards (4,653 patients) from 2006 to 2011 (55). Recent data from the HIPSTA surgical training ward in Germany, as published by Kuner et al., revealed no substantial differences in the rate or severity of postoperative complications between 232 training ward patients and 465 conventional ward patients (58). Notably, the training ward saw fewer reoperations, demonstrating a variance in surgical intervention frequencies between the two settings. Hansen et al. evaluated the quality of life in 62 patients from a Danish orthopedic training ward and 72 from conventional wards 3 months post-hospital stay, finding no significant differences in outcomes between the two groups (57).

Comparable mortality rates were observed between the A-STAR group and conventional wards, with most deaths in both contingents attributed to the palliative status of underlying conditions. This aligns with Hallin et al. and Kuner et al., who reported no significant mortality rate differences in their cohorts (55, 58).

These findings reinforce the safety and reliability of patient care in training wards, ensuring that patients can confidently receive treatment in these environments. A prevalent limitation across all studies, the present study included, is the need for more controlled patient randomization. Nevertheless, existing data suggests that implementing such randomization would not negatively impact patient outcomes. Further research, especially focusing on the quality of patient transitions to post-inpatient sectors and family care quality, is imperative to understand the impacts of interprofessional training wards on patient care and outcomes thoroughly.

## Data availability statement

The raw data supporting the conclusions of this article will be made available by the authors, without undue reservation.

## Ethics statement

The studies involving humans were approved by Ethikkommission bei der Universität Regenburg Universität Regensburg, 93040 Regensburg (20-1805_1-101). The studies were conducted in accordance with the local legislation and institutional requirements. The participants provided their written informed consent to participate in this study.

## Author contributions

SS-H: Conceptualization, Data curation, Formal analysis, Project administration, Supervision, Validation, Visualization, Writing – original draft, Writing – review & editing. EA: Data curation, Investigation, Writing – review & editing. MMe: Data curation, Investigation, Writing – review & editing. SA-F: Conceptualization, Methodology, Writing – review & editing. KR: Conceptualization, Methodology, Writing – review & editing. SR: Writing – original draft, Writing – review & editing. BM: Writing – review & editing. AM: Writing – review & editing. CK: Supervision, Writing – review & editing. SS: Writing – review & editing. MMü: Conceptualization, Funding acquisition, Methodology, Project administration, Resources, Supervision, Validation, Visualization, Writing – original draft, Writing – review & editing.

## Supplementary material

SUPPLEMENTARY DATA SHEET 1 German questionnaire.

SUPPLEMENTARY DATA SHEET 2 Questionnaire in English translation.
