# Supplementary material for: Evaluating the impact of interprofessional training wards on patient satisfaction and clinical outcomes: a mixed-methods analysis
Source: Front Med (Lausanne). 2024 Feb 20;11:1320027. doi: 10.3389/fmed.2024.1320027 (PMC10912604; doi:10.3389/fmed.2024.1320027)
Supplement: SUPPLEMENTARY DATA SHEET 1 — German questionnaire. [file Data_Sheet_1.PDF]

1. Allgemeines zu Ihrem Aufenthalt

	zutreffend	eher zutreffend	eher nicht zutreffend	nicht zutreffend	weiß ich nicht
1.1 Ich habe mich auf der Station gut aufgehoben gefühlt.	O	O	O	O	O
1.2 Ich habe einen Unterschied in der Zusammenarbeit des Teams im Vergleich zu anderen Stationen festgestellt.	O	O	O	O	O
1.3 Ich bin mit dem Ablauf der Visiten zufrieden.	O	O	O	O	O
1.4 Ich bin mit der Atmosphäre der Visiten zufrieden.	O	O	O	O	O
1.5 Ärzte und Pflegekräfte arbeiten Hand in Hand.	O	O	O	O	O
1.6 Absprachen zwischen Ärzten und Pflegekräften untereinander haben gut funktioniert.	O	O	O	O	O
1.7 Die einzelnen Behandlungsmaßnahmen waren gut aufeinander abgestimmt.	O	O	O	O	O
1.8 Ich habe mit dem Team (Ärzte/PJ-Studenten/ Pflegekräfte/ Pflegeschüler) gemeinsam die weitere Versorgung festgelegt und bin mit dem Ergebnis zufrieden.	O	O	O	O	O
1.9 Mit der Art und Weise, wie meine Behandlung im Rahmen dieses Klinikaufenthaltes besprochen worden ist, bin ich zufrieden.	O	O	O	O	O
1.10 Die Zusammenarbeit von ärztlichen und pflegerischen Mitarbeitenden war besser als auf anderen Stationen.	O	O	O	O	O
1.11 Das Team hat einen harmonischen Eindruck gemacht.	O	O	O	O	O
1.12 Ich bin mit der Aufklärung über Behandlung und Verlauf meiner Krankheit zufrieden.	O	O	O	O	O
1.13 Meine Ängste und Sorgen wurden durch das Team berücksichtigt.	O	O	O	O	O
1.14 Das Team hat mir geholfen, alle Informationen zu verstehen.	O	O	O	O	O
1.15 Das Team hat mir alle meine Fragen beantwortet.	O	O	O	O	O
1.16 Das Team hat verstanden, was für mich wichtig ist.	O	O	O	O	O
1.17 Die Zusammenarbeit des Teams hat sich positiv auf mein Wohlbefinden ausgewirkt.	O	O	O	O	O
1.18 Mit der Einbindung meiner Angehörigen in Gespräche bin ich zufrieden.	O	O	O	O	O
1.19 Ich bin mit der Behandlung meiner Beschwerden zufrieden.	O	O	O	O	O
1.20 Ich würde meinen Freunden/Familie eine Behandlung auf der Station empfehlen.	O	O	O	O	O
1.21 Ich bin mit der allgemeinen Stimmung auf der Station zufrieden.	O	O	O	O	O

© Prof. Dr. Martina Müller-Schilling, Dr. Sophie Schlosser, Dr. Sheila Albaladejo - Fuertes, Kirstin Ruttmann

2. **Wie bewerten Sie die Organisation?**

	sehr gut	eher gut	eher schlecht	sehr schlecht	kann ich nicht beurteilen
2.1 bei der Aufnahme?	O	O	O	O	O
2.2 bei Untersuchungen?	O	O	O	O	O
2.3 bei pflegerischen Maßnahmen?	O	O	O	O	O

3. **Wie bewerten Sie Ihr Behandlungsteam in folgenden Bereichen?**

	sehr gut	eher gut	eher schlecht	sehr schlecht	kann ich nicht beurteilen
3.1 Fachwissen und Kompetenz	O	O	O	O	O
3.2 Gesprächsführung (Kommunikation)	O	O	O	O	O
3.3 Sicheres Auftreten	O	O	O	O	O
3.4 Einfühlungsvermögen (Empathie)	O	O	O	O	O

4. **Wie bewerten Sie die betreuenden Pflegeauszubildenden in den folgenden Bereichen?**

	sehr gut	eher gut	eher schlecht	sehr schlecht	kann ich nicht beurteilen
3.1 Fachwissen und Kompetenz	O	O	O	O	O
3.2 Gesprächsführung (Kommunikation)	O	O	O	O	O
3.3 Sicheres Auftreten	O	O	O	O	O
3.4 Einfühlungsvermögen (Empathie)	O	O	O	O	O

5. **Wie bewerten Sie die betreuenden Medizinstudenten in den folgenden Bereichen?**

	sehr gut	eher gut	eher schlecht	sehr schlecht	kann ich nicht beurteilen
3.1 Fachwissen und Kompetenz	O	O	O	O	O
3.2 Gesprächsführung (Kommunikation)	O	O	O	O	O
3.3 Sicheres Auftreten	O	O	O	O	O
3.4 Einfühlungsvermögen (Empathie)	O	O	O	O	O

6. **Wie ging es Ihnen gesundheitlich während Ihres Aufenthalts auf der Station?**

	sehr gut	eher gut	eher schlecht	sehr schlecht	kann ich nicht beurteilen
6.1 Am Tag der Aufnahme	O	O	O	O	O
6.2 Am Tag der Befragung	O	O	O	O	O

7. **Lob, Kritik und Anregung**

7.1 Was fanden Sie auf der Station besonders gut?
7.2 Was könnten wir Ihrer Meinung nach noch besser machen?

© Prof. Dr. Martina Müller-Schilling, Dr. Sophie Schlosser, Dr. Sheila Albaladejo - Fuertes, Kirstin Ruttmann

8. Welcher Altersgruppe gehören Sie an?	O 18 bis 24 Jahre<br>O 25 bis 34 Jahre<br>O 35 bis 50 Jahre<br>O 51 bis 70 Jahre<br>O über 70 Jahre
9. Wie viele Tage waren Sie bei uns?	O 1-3 Tage<br>O 4-7 Tage<br>O 8-14 Tage<br>O mehr als 14 Tage

Das Team dankt Ihnen für die Unterstützung und wünscht Ihnen für Ihre Gesundheit weiterhin alles Gute!

© Prof. Dr. Martina Müller-Schilling, Dr. Sophie Schlosser, Dr. Sheila Albaladejo - Fuertes, Kirstin Ruttmann
