# Supplementary material for: Evaluating the impact of interprofessional training wards on patient satisfaction and clinical outcomes: a mixed-methods analysis
Source: Front Med (Lausanne). 2024 Feb 20;11:1320027. doi: 10.3389/fmed.2024.1320027 (PMC10912604; doi:10.3389/fmed.2024.1320027)
Supplement: SUPPLEMENTARY DATA SHEET 2 — Questionnaire in English translation. [file Data_Sheet_2.PDF]

1. General information about your stay

	applicable	rather applicable	rather not applicable	not applicable	I don’t know
1.1 I felt well cared for in the interprofessional ward.	O	O	O	O	O
1.2 I have noticed a positive difference in the team's cooperation compared to other wards	O	O	O	O	O
1.3 I am satisfied with the way the ward rounds are conducted.	O	O	O	O	O
1.4 I am satisfied with the atmosphere of the ward rounds.	O	O	O	O	O
1.5 Doctors and nurses work hand in hand.	O	O	O	O	O
1.6 Arrangements between physicians and nurses worked well.	O	O	O	O	O
1.7 The individual treatment measures were well coordinated.	O	O	O	O	O
1.8 I worked with the team (physicians/students in the final year/nurses/training nurses) to determine further care and am satisfied with the outcome.	O	O	O	O	O
1.9 I am satisfied with the way my treatment was discussed during this hospital stay.	O	O	O	O	O
1.10 The cooperation between medical and nursing staff was better than in other wards.	O	O	O	O	O
1.11 The team made a harmonious impression.	O	O	O	O	O
1.12 I am satisfied with the information about the treatment and course of my disease.	O	O	O	O	O
1.13 My fears and concerns were taken into account by the interprofessional team.	O	O	O	O	O
1.14 The team helped me to understand all the information.	O	O	O	O	O
1.15 The team answered all my medical questions.	O	O	O	O	O
1.16 The team understood what was important to me.	O	O	O	O	O
1.17 The team's collaboration has had a positive impact on my well-being.	O	O	O	O	O
1.18 I am satisfied with the involvement of my relatives in discussions.	O	O	O	O	O
1.19 I am satisfied with the treatment of my medical complaints.	O	O	O	O	O
1.20 I would recommend treatment on the ward to my friends/family.	O	O	O	O	O
1.21 I am satisfied with the general atmosphere on the ward.	O	O	O	O	O

© Prof. Dr. Martina Müller-Schilling, Dr. Sophie Schlosser, Dr. Sheila Albaladejo - Fuertes, Kirstin Ruttmann

2. **How would you rate the organisation?**

	very good	rather good	rather bad	very bad	I cannot judge
2.1 during admission?	O	O	O	O	O
2.2 during examinations?	O	O	O	O	O
2.3 during nursing measures?	O	O	O	O	O

3. **How would you rate your medical team in the following areas?**

	very good	rather good	rather bad	very bad	I cannot judge
3.1 Knowledge and competence	O	O	O	O	O
3.2 Communication	O	O	O	O	O
3.3 Professional appearance	O	O	O	O	O
3.4 Empathy	O	O	O	O	O

4. **How would you rate your nursing trainees in the following areas?**

	very good	rather good	rather bad	very bad	I cannot judge
3.1 Knowledge and competence	O	O	O	O	O
3.2 Communication	O	O	O	O	O
3.3 Professional appearance	O	O	O	O	O
3.4 Empathy	O	O	O	O	O

5. **How would you rate your medical students in the following areas?**

	very good	rather good	rather bad	very bad	I cannot judge
3.1 Knowledge and competence	O	O	O	O	O
3.2 Communication	O	O	O	O	O
3.3 Professional appearance	O	O	O	O	O
3.4 Empathy	O	O	O	O	O

6. **How was your health during your stay on the ward?**

	very good	rather good	rather bad	very bad	I cannot judge
6.1 On the day of admission	O	O	O	O	O
6.2 On the day of the interview	O	O	O	O	O

7. **Feedback**

7.1 What did you find particularly good on the ward?
7.2 What do you think we could do better?

© Prof. Dr. Martina Müller-Schilling, Dr. Sophie Schlosser, Dr. Sheila Albaladejo - Fuertes, Kirstin Ruttmann

8. Which age group do you belong to?	O 18 to 24 years<br>O 25 to 34 years<br>O 35 to 50 years<br>O 51 to 70 years<br>O above 70 years
9. How many days were you with us?	O 1-3 days<br>O 4-7 days<br>O 8-14 days<br>O more than 14 days

The team thanks you for your support and wishes you continued good health!

© Prof. Dr. Martina Müller-Schilling, Dr. Sophie Schlosser, Dr. Sheila Albaladejo - Fuertes, Kirstin Ruttmann
